# Continuous weeklong measurements of indoor particle levels in a Minnesota Tribal Casino Resort

**DOI:** 10.1186/s12889-016-3553-4

**Published:** 2016-08-24

**Authors:** Zheng Zhou, David Bohac, Raymond G. Boyle

**Affiliations:** 1 Department of Environmental Health Sciences, Mailman School of Public Health, Columbia University, 722 West 168th Street, 11th Floor, New York, NY 10032 USA; 2 Center for Energy and Environment, 212 Third Avenue North, Suite 560, Minneapolis, MN 55401 USA; 3 Clearway Minnesota SM, 8011 34th Avenue South, Suite 400, Minneapolis, MN 55425 USA

**Keywords:** Secondhand smoke, Fine particulate matter, Real-time monitoring, Indoor air pollution, Tribal casino

## Abstract

**Background:**

Secondhand smoke (SHS) exposure for workers and patrons in hospitality venues is a persistent and significant public health concern. We designed this study to provide a comprehensive assessment of SHS exposure inside an Indian Tribal Casino in Minnesota.

**Methods:**

Real-time fine particulate matter (PM_2.5_) concentrations were measured at multiple locations for up to 7 days. The field monitoring provided information on the day of week and time of day variation of SHS exposure, as well as comparisons between smoking and non-smoking areas.

**Results:**

Indoor PM_2.5_ level was nearly 13 times the concurrent outdoor PM_2.5_ level. Gaming floor hourly PM_2.5_ level was highest on Saturday night, averaged at 62.9 μg/m^3^. Highest PM_2.5_ concentration was observed in smoking-permitted employee break room, reaching 600 μg/m^3^. PM_2.5_ readings in non-smoking sections exhibited same temporal pattern as the readings in smoking sections.

**Conclusions:**

The results show that indoor concentration of PM_2.5_ is substantially higher than the outdoor level, posing health risks to casino workers and patrons. SHS can migrate into adjacent non-smoking areas very quickly. The casino’s ventilation system did not fully eliminate SHS. A completely smoke-free casino would be the only way to fully protect non-smoking patrons and employees from the dangers of tobacco smoke.

**Electronic supplementary material:**

The online version of this article (doi:10.1186/s12889-016-3553-4) contains supplementary material, which is available to authorized users.

## Background

Secondhand smoke (SHS) is a mixture of gases and particles that consist of more than 7,000 chemical compounds. Previous U.S. Surgeon General reports confirm that SHS is associated with an increased risk of lung cancer, coronary heart disease, respiratory illness, and other diseases [1, 2]. Studies have shown that even short-term exposure to SHS can be harmful. In a controlled laboratory setting, Junker et al. [3] reported that odor sensation can be perceived at low levels of SHS exposure (0.6–1.4 μg/m^3^), and that people start to feel eye, nasal, and throat irritations at 4.4 μg/m^3^. In another study, Pope et al. [4] found a decline of 12 % in heart rate variability after 1.75-h exposure to 53 μg/m^3^ of suspended respirable particles from SHS.

Indoor smoking bans have been introduced by many states and countries to protect workers and the public from SHS exposure. As of July 1, 2016, 41 states in the U.S. have smoke-free laws in non-hospitality workplaces, including restaurants and/or bars [5]. However, many states exempt casino venues from their indoor smoking bans. In addition, American Indian casinos are controlled by tribal sovereign nations and are not subject to prevailing smoking bans.

Workers and patrons in smoking-allowed casinos are often exposed to elevated level of SHS, which can be determined by measuring concentrations of tobacco smoke components such as nicotine and fine particles (<2.5 μm in diameter; PM_2.5_). A study of 66 U.S. casinos found that short-term (0.5–1 h) average PM_2.5_ concentrations had a geometric mean of 53.8 μg/m^3^ (range 18.5–205 μg/m^3^), more than 10 times the outdoor levels (4.3 μg/m^3^, range 0.26–29.7 μg/m^3^) [6]. Siegel and Skeer [7] reported that nicotine concentrations in casino venues were 2.4–18.5 times higher than in offices or residences, and 1.5–11.7 times higher than in restaurants. In a review of recently published studies on SHS exposure and health effects in casinos, Babb et al. [8] concluded that SHS is a significant, preventable health risk for workers and patrons in casinos that allow smoking.

There are currently 18 tribal casinos in Minnesota. One of them, Shooting Star Casino, is owned and operated by the White Earth Nation, which is located in northwestern Minnesota and is one of six member reservations of the Minnesota Chippewa. With an enrolled population of more than 20,000, White Earth is the largest tribe in Minnesota. Since 2003, the Tribal Health Education program has organized a Tobacco Coalition to address tobacco use and SHS exposure among members of the White Earth Nation. In September 2012, the Shooting Star Casino Facilities Manager, who is a member of the Tobacco Coalition, attended the Clean & Healthy Tribal Casinos workshop, held by U.S. EPA in Grand Portage, Minnesota. Following the conference, the coalition and the health department obtained approval from tribal leadership to pursue a study of SHS exposure in the casino and other indoor spaces. The White Earth Health Department approached ClearWay Minnesota, asking for support and technical assistance in assessing SHS exposure at the Shooting Star Casino. ClearWay Minnesota is a nonprofit tobacco control organization that has provided funding to White Earth to support smoke-free events and spaces in the community. As part of this process, the Center for Energy and Environment, a Minnesota nonprofit with a research department that conducts building energy and indoor air quality (IAQ) studies, was invited to perform an IAQ study at the Shooting Star Casino. Field monitoring was conducted at the end of December 2013.

Although a number of studies have measured air quality inside casinos that permit smoking, almost all of these studies have been based on limited access by time and location [6, 7, 9, 10]. Specifically, these studies have been based on: 1) a short measurement period (0.5–1 h); 2) a single visit to venues during peak occupancy periods; 3) monitoring limited to only one or two locations; and 4) no information on the casino ventilation system.

The study reported here is the result of the collaboration between White Earth and ClearWay Minnesota to address shortcomings of previous studies and to assess the contribution of SHS to indoor PM_2.5_ in multiple locations of a tribal casino resort. The specific research objectives were: 1) to determine the proportion of casino patrons smoking and to contrast this with the Minnesota state-wide smoking prevalence; 2) to contrast indoor air quality with outside air; 3) to contrast indoor air quality in smoking-permitted areas compared to non-smoking areas; and 4) to determine if ventilation and separation of the non-smoking area had any effect on indoor air quality.

## Methods

### Study site

The shooting star casino has a gaming floor of approximately 72,000 square-feet, including nearly 1,100 slot machines, 12 blackjack tables, and a 365-sear bingo hall. The gaming floor is divided into smoking and non-smoking areas. There is no physical barrier (e.g. door or wall) between the two areas. The size of the designated non-smoking gaming area is about 25 % of the gaming floor. There are four on-site restaurants, including a fine dining restaurant, 2 ONE 8, and casual dining options at the Whispering Winds, Deli Cafe, and Traditions Buffet. 2-one-8 is fully enclosed from gaming area and has a door, while the other three restaurants are semi-enclosed and have open doorway between the restaurants and the gaming area. One of our focus areas is employee break room, which has smoking and non-smoking sections. A wall with an open door separates the two sections. There are five air handlers serving the casino. Traditions Buffet and 2 ONE 8 each has its own air handler. One air handler serves the non-smoking gaming floor. The other two air handlers serve the smoking gaming floor as well as the Whispering Winds and Deli Café.

### Study design

Air quality monitoring was designed to provide comprehensive information on the day of week and time of day variation of fine particle (PM_2.5_) exposure as well as spatial differentiation. We conducted one week long monitoring of PM_2.5_ levels at multiple locations inside the casino. The monitoring locations included: 1) outdoor air and return air from the gaming floor (7 days), 2) fixed sites on the gaming floor (5 days), 3) restaurants (~30 min), and 4) employee break room (~10 h).

Outdoor air PM_2.5_ concentration was measured in a fresh air supply duct, while indoor PM_2.5_ level was measured in a return air duct for the smoking gaming area. In addition, we measured surface level PM_2.5_ concentrations at fixed sites in smoking and non-smoking gaming areas. Measurements in restaurants were short-term, taken for about 30 min during dinnertime. Three locations were monitored in or near each restaurant. One was located right outside the restaurant, the second was inside but close to the entrance, and the third one was further inside near dining tables. The employee break room is divided into smoking and non-smoking sections that are connected by an open doorway. PM_2.5_ levels were measured simultaneously in the two sections between 3 PM and 8 AM on one of the monitoring days.

In addition to the PM_2.5_ monitoring, the number of casino patrons and active smokers were counted between 7 and 9 PM on 3 days. An investigator walked through the entire smoking-permitted gaming area and counted the number of active smokers and total patrons. Multiple sets of counts were conducted and each count took about 20 min. The active smoking prevalence (total number of active smokers/total number of casino patrons × 100 %) is reported.

### Study instrument

SidePak™ AM510 Aerosol Monitors (TSI, Inc, St. Paul, MN) were used for continuous, real-time monitoring of PM_2.5_. The unit has a laser photometer that uses a 90° light scattering system with a 670-nm laser diode to indicate the particle total mass concentration. The manufacturer-specified minimum resolution is 1 μg/m^3^ with a particle measurement range from 0.1 to 10 μm. The zero stability is ±1 μg/m^3^ over 24 h, and it has a temperature coefficient of +0.5 μg/m^3^ per °C. For the monitoring conducted for this study the logging interval was set to 1 min and the unit was fitted with a 2.5 μm 50 % cut point impactor with the flow rate adjusted to 1.7 l per minute. PM concentrations measured by laser photometry method are subject to error due to the use of specific aerosols for factory calibration, whose characteristics (e.g., shape, size, density and refractive index) may differ from those in our study. The manufacturer recommends that users determine the calibration factor for the specific monitoring application. A calibration factor of 0.31 was used based on previous studies that reported factors from 0.295 to 0.328 for SHS [11–13]. In order to make direct comparison we applied the calibration factor (0.31) to both indoor and outdoor measurements. In general, outdoor PM_2.5_ levels are very low and their absolute values are therefore less affected by the calibration factor.

### Data analysis

PM_2.5_ data were averaged at 1-min interval. Mean PM_2.5_ concentrations are reported to compare SHS exposure at different locations and different time in the casino. T-test was used to test the difference between the means at a statistical significance level of 0.05. All analyses were conducted using the open-source statistical analysis package R version 3.0.

## Results

### Observation data on active smokers

Consistent with gaming establishments, the number of patrons was greater on the weekends (see Table 1). There were 57 % more patrons on the Friday evening (330 people) than the previous day (210 people), and 104 % more than the following Thursday (162 people). On average, active smokers accounted for 13–5 % of casino patrons in the smoking-permitted gaming area.

**Table 1:** Observation data on the number of casino patrons and active smokers in the smoking-permitted gaming area during peak hours

Date	Time^a^	# Patrons	# Active smokers	% Active smokers
Thu Dec. 12	7:40–8:40 pm	210	28	13.3 %
Fri Dec. 13	7:40–8:40 pm	330	43	13.0 %
Thu Dec. 19	7:30–8:10 pm	162	25	15.4 %

^a^Counts recorded every 20 min, average numbers were reported

### Outdoor vs. indoor air quality

**Fig. 1:**
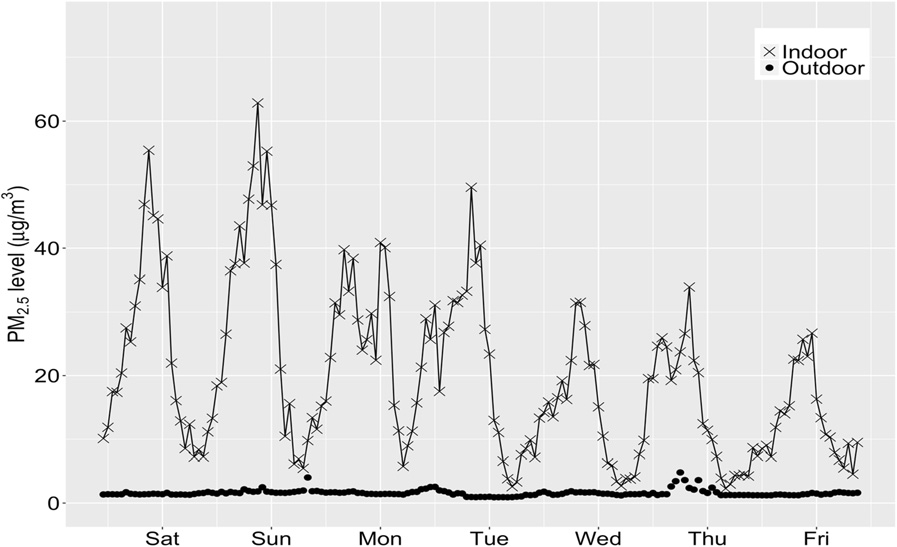
Time series of hourly PM_2.5_ concentrations measured in fresh air supply duct (outdoor air) and return air duct (indoor air) for the smoking gaming area

In the casino the mean level of indoor PM_2.5_ during the weeklong monitoring period was 20.1 μg/m^3^, nearly 13 times of the average outdoor PM_2.5_ level (1.6 μg/m^3^). This outdoor level is consistent with low concentrations for the rural northern Minnesota location of the resort. Figure 1 presents a clear diurnal pattern of indoor PM_2.5_ concentration, with the indoor level rising in the early morning, reaching a peak between 8 and 9 PM, and then gradually declining after midnight to a level close to the outdoor level. This pattern was similar to the daily business cycle for the casino where late night is the busiest time. Differences in PM_2.5_ level are also found between weekend and weekday, which matches our observation of the total number of casino patrons and active smokers. 24-h average indoor PM_2.5_ levels ranged from 13.4 μg/m^3^ (Thursday) to 28.4 μg/m^3^ (Saturday). Peak PM_2.5_ concentration (1-h maximum) during the weeklong monitoring ranged from 26.7 μg/m^3^ (Thursday night) to 62.9 μg/m^3^ (Saturday night). All these results suggest that smoking is the major pollution source inside the casino.

### Smoking vs non-smoking areas

**Fig. 2:**
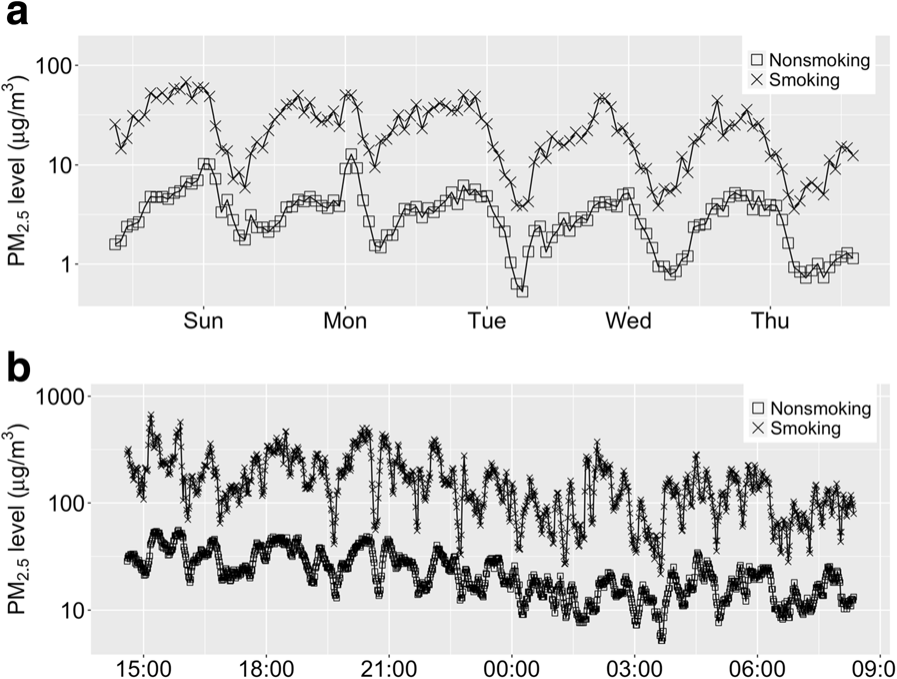
Concurrent PM_2.5_ concentrations measured in smoking-permitted and non-smoking areas in the casino: **a** hourly average PM_2.5_ concentrations on gaming floor; and **b** minute average PM_2.5_ concentrations in employee break room

Air quality monitoring was conducted simultaneously in the adjacent smoking and non-smoking sections of the gaming floor and the employee break room. Figure 2a shows that PM_2.5_ concentrations measured in smoking and non-smoking sections of the gaming floor had similar temporal patterns. The correlation coefficient is 0.58 (0.74 after log-transformation) for the PM_2.5_ levels in the smoking and non-smoking areas. In comparison, the correlation between the levels in the non-smoking gaming area and the outdoor air was only 0.17. The average PM_2.5_ level in the smoking gaming area over the 5-day monitoring was 24.7 μg/m^3^, while the average level in the non-smoking gaming area was 3.4 μg/m^3^, which is higher than the outdoor level (1.6 μg/m^3^). The difference between smoking and non-smoking area PM_2.5_ levels is statistically significant (*P* < 0.001).

Figure 2b displays the time series of PM_2.5_ levels inside the employee break room. The 1-min readings for the smoking section exhibit many short-interval “spikes”, and the non-smoking section reading closely resembles the pattern at a lower level. A high correlation (correlation coefficient: 0.78) was observed between the two measurement locations. The PM_2.5_ level in the smoking section reached a peak of 670 μg/m^3^ and averaged 164 μg/m^3^, statistically significantly (*p* < 0.001) higher than the level in the non-smoking section (23 μg/m^3^) and nearly 5 times the EPA 24-h standard. This strongly indicates SHS rapidly penetrates from the smoking area into the adjacent non-smoking area.

### The effect of ventilation and physical isolation on eliminating SHS in non-smoking areas

**Fig. 3:**
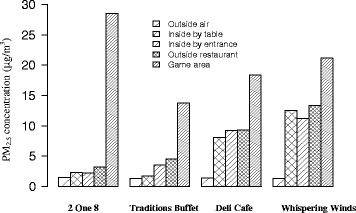
Average PM_2.5_ levels in restaurants, gaming area and outside air around dinnertime

An argument is often made that using higher ventilation rates can remove particles from smoking. We measured PM_2.5_ levels outside, at the entrance, and inside the 4 non-smoking on-site restaurants to examine how well the casino’s ventilation system and the physical isolation of the restaurant areas from smoking-permitted areas protects people in non-smoking areas. Figure 3 compares average PM_2.5_ levels in or near the restaurants, and concurrent PM_2.5_ levels in outside air and return air from the gaming area. For all 4 restaurants, indoor PM_2.5_ levels were higher than the concurrent outdoor levels, indicating that ventilation and isolation was not able to eliminate SHS exposure.

The difference in the gaming area PM_2.5_ level is due to different measurement times. In general, lower PM_2.5_ levels were observed in 2 One 8 and Traditions Buffet compared to the Deli Café and Whispering Winds. Inside-by-table PM_2.5_ levels in 2 One 8 and Traditions Buffet were 2.3 and 1.7 μg/m^3^, about 1/10 of the concurrent PM_2.5_ levels in the smoking gaming area. In contrast, the inside-by-table PM_2.5_ levels in the Deli Cafe and Whispering winds were 8.1 and 12.6 μg/m^3^, nearly 1/2 of the concurrent gaming area levels. The results suggest that the ventilation systems and the degree of separation for 2 One 8 and Traditions Buffet are more effective in removing SHS than with the other two restaurants. Indeed, 2 One 8 and Traditions Buffet each has its own air handler, while Deli Café and Whispering Winds share air handlers with the gaming area, therefore receiving SHS recycled from ventilation system.

## Discussion

To our knowledge, this study is the first to provide a comprehensive assessment of secondhand smoke exposure in a Minnesota tribal casino, and it may motivate other casinos to take actions to protect the health of workers and patrons.

The observed percent of active smokers in the casino is similar to the active smoker prevalence reported in 36 California Indian casinos of 11 % (range, 5–25 %) [9], and the prevalence reported in 7 Reno casinos of 9.3 % (range, 7–12 %) [6]. Klepeis et al. [10] found a somewhat lower active smoker proportion of 7 % (range, 5–10 %) in 11 southern California casinos. The observed percent of active smokers is also similar to the smoking prevalence of Minnesota adults (14.4 %, Minnesota Adult Tobacco Survey) [14]. However, previous studies found that the number of smokers is about three times the number of active smokers in gaming establishments [15, 16]. This suggests the percent of smokers inside the casino might be higher than the state prevalence of smoking.

A number of studies have reported higher PM_2.5_ concentrations inside casinos than our study. Jiang et al. [9] found that the mean PM_2.5_ concentration over 0.5–1 h in smoking sections was 63 μg/m^3^ (range 18–183 μg/m^3^) in 36 California Indian casinos. Repace et al. [6] reported a mean of 45.2 μg/m^3^ (95 % CI 37.7–52.7 μg/m^3^) in 21 Reno and Las Vegas smoking casinos. The main difference is that previous studies only measured PM_2.5_ level for a short period (30 mins to a few hour) during casino peak times. The Peak PM_2.5_ concentrations in our study are comparable to the findings of those studies. It is also worth noting that our study was conducted during a non-holiday week, thus it is expected that the number of casino patrons and smokers would be higher during busier times (e.g. Christmas, New Year), likely resulting in higher SHS exposure inside the casino. Although daily average PM_2.5_ levels during the monitoring week in the gaming area were lower than 35 μg/m^3^, the U.S. EPA 24-h ambient PM_2.5_ standard, 24 out of 167 h had hourly average PM_2.5_ concentration above the standard.

The study found that fine particle concentrations varied substantially across different locations inside the casino, highest in the employee break room and lowest inside the non-smoking restaurant 2 One 8. The SHS exposure in two of the non-smoking restaurants was reduced by 90 % due to the use of separate air handlers. In contrast, the non-smoking restaurants that share an air handler with the smoking gaming area led to SHS being drawn into non-smoking areas through the ventilation system.

In March 2014, we presented the study results to the tribal council of White Earth Nation. We illustrated that a completely smoke-free casino would be the only way to fully protect non-smoking patrons and employees from the dangers of tobacco smoke. After reviewing the results, the council decided to make the employee break room smoke-free and created a separate outdoor space for employees to smoke.

The most common concern raised by the gaming industry is that smoking bans will lead to less gaming and therefore lower revenue. Previous studies that have examined the economic impact of smoke-free policies on casinos have found mixed results [8]. Recently, Klepeis et al. [17] reported a case where a California tribal casino switched from 100 % smoke-free to 70 % smoke-free due to reduced revenue. However, the study suggests that the revenue loss may be due to other factors besides smoke-free policies. Further research is needed to address financial concern on the adoption of smoke-free policy in casinos.

## Conclusions

Our collaboration with Shooting Star casino enabled us to explore temporal and spatial variability of SHS exposure inside a casino and dining facilities. The results suggest that indoor concentration of PM_2.5_ is substantially higher than the outdoor level, posing health risks to casino workers and patrons. The results also demonstrate that SHS can migrate into adjacent non-smoking areas very quickly, especially when there is no physical barrier. The casino’s ventilation system did not fully eliminate SHS.

## Additional file

Additional file 1: Supplementary dataset. (XLSX 739 kb)

## Abbreviations

SHS: Secondhand smoke
PM_2.5_: Fine particulate matter
IAQ: Indoor air quality
